# The Impact of Mushroom Management on Nurses’ Job Satisfaction: The Mediating Role of Organizational Identification

**DOI:** 10.1155/jonm/8888440

**Published:** 2026-04-30

**Authors:** Delal Aydın, Özcan Demir, Ömer Karakoyun, Nur Ecer, Erhan Ayhan

**Affiliations:** ^1^ Department of Health Management, Fırat University Faculty of Economics and Administrative Sciences, Elazığ, Türkiye; ^2^ Department of Business Administration, Fırat University Faculty of Economics and Administrative Sciences, Elazığ, Türkiye; ^3^ Department of Dermatology and Venereology, Gazi Yaşargil Training and Research Hospital, Diyarbakır, Türkiye; ^4^ Department of Dermatological and Venereal Diseases, Private Dermatology Practice, Diyarbakır, Türkiye; ^5^ Department of Dermatological and Venereal Diseases, Dicle University Faculty of Medicine, Diyarbakır, Türkiye, dicle.edu.tr

**Keywords:** healthcare leadership, job satisfaction, mediation analysis, mushroom management, nurses, nursing management, organizational identification, Türkiye

## Abstract

**Background:**

Mushroom management, a leadership style characterized by withholding information, excluding employees from strategic processes, and maintaining top‐down control, has been linked to adverse organizational outcomes. However, its impact on nurses and the psychological mechanisms through which this influence operates remain underexamined.

**Purpose:**

This study examines the direct and indirect effects of mushroom management on nurses’ job satisfaction, with organizational identification (OI) as the mediating variable, guided by affective events theory and social identity theory.

**Methods:**

A cross‐sectional survey was conducted with 317 nurses employed in private hospitals in Diyarbakır, Türkiye. Data were collected using validated Turkish versions of the Mushroom Management Scale (Birincioğlu and Tekin, 2018), Job Satisfaction Scale (Judge et al., 2008; Keser and Bilir, 2019), and Organizational Identification Scale (Mael, 1988; Tüzün and Özdoğan, 2006). All scales use a five‐point Likert format (1 = *strongly disagree* to 5 = *strongly agree*). Confirmatory factor analysis confirmed good model fit (*χ* ^2^/d*f* = 2.21, CFI = 0.95, RMSEA = 0.062). Mediation was tested using Hayes’ PROCESS macro (Model 4) in SPSS 23.0 with 5000 bootstrap samples.

**Results:**

Mushroom management had significant negative effects on OI (standardized *β* = −0.46, *p* < 0.001) and job satisfaction (standardized *β* = −0.21, *p* = 0.001). OI positively predicted job satisfaction (standardized *β* = 0.48, *p* < 0.001). Mediation analysis confirmed a significant indirect effect (standardized *β* = −0.22, 95% CI [−0.31, −0.14]), with OI accounting for 51% of the total effect indicating partial mediation.

**Conclusions:**

Mushroom management is associated with lower OI and job satisfaction among nurses, with OI serving as a key identity‐based psychological mechanism. Healthcare leaders should prioritize transparent communication, participatory decision‐making, and identity‐affirming leadership practices to enhance nurse well‐being and retention.

**Implications for Nursing Management:**

Findings highlight the need for structured information‐sharing protocols, mushroom‐management–specific leadership training, and shared governance structures in private hospital settings. Periodic organizational climate monitoring using validated instruments is recommended.

## 1. Introduction

Healthcare organizations operate in highly complex, dynamic, and resource‐constrained environments where leadership styles significantly influence workforce performance, job satisfaction, and organizational commitment. Within this context, the nursing profession occupies a critical position, as nurses constitute the largest segment of the healthcare workforce and directly impact patient outcomes, safety, and service quality [1, 2]. Leadership approaches that fail to provide transparent communication, shared decision‐making, and access to essential information can adversely affect nurses’ psychological well‐being and professional engagement.

One such detrimental leadership approach is mushroom management, a multidimensional construct referring to the intentional withholding of information from employees, excluding them from decision‐making processes, and maintaining top‐down control driven by fear of losing power [3]. The term derives from the metaphor of keeping mushrooms “in the dark and feeding them manure,” reflecting a management philosophy in which employees are deliberately kept uninformed and marginalized. This style has been associated with decreased trust, weakened organizational identification (OI), and reduced job satisfaction across organizational settings [4, 5].

Although mushroom management shares surface‐level similarities with several adjacent leadership constructs, it is conceptually distinct. Authoritarian leadership centers on dominance and the punishment of dissent, whereas mushroom management operates primarily through omission and passivity rather than overt intimidation. Knowledge hiding is an individual‐level behavior rather than a managerial style. Abusive supervision involves sustained hostile behaviors, while the harm inflicted by mushroom management is largely passive, stemming from deliberate information withholding rather than active mistreatment. Informational injustice describes an employee’s fairness perception, whereas mushroom management is the leadership practice that may give rise to such perceptions. Table 1 summarizes these distinctions.

**TABLE 1:** Conceptual comparison of mushroom management and adjacent leadership constructs.

Construct	Core definition	Primary focus	Overlap with MM	Key distinction from MM
Mushroom management	Intentional withholding of information, exclusion from decision‐making, top‐down control	Information opacity + power retention + exclusion	—	Multidimensional: combines information restriction, fear of power loss, and lack of participation
Authoritarian leadership	Leader demands absolute obedience and punishes dissent	Dominance and control	Top‐down control	MM does not necessarily involve punishment or intimidation
Knowledge hiding	Deliberate concealment of requested information	Information concealment (individual behavior)	Information restriction	MM is a managerial style/structure; knowledge hiding is an individual behavior
Abusive supervision	Sustained hostile verbal and nonverbal behaviors by supervisors	Hostile, demeaning treatment	Power imbalance	MM lacks overt hostility; harm is passive (through omission, not commission)
Informational injustice	Perceived unfairness in adequacy and timeliness of information shared	Fairness perceptions about information	Inadequate information sharing	MM is a leadership style; informational injustice is an employee perception/outcome

Abbreviation: MM, mushroom management.

Two complementary theoretical frameworks guide this study. Affective events theory (AET), proposed by Weiss and Cropanzano [6], posits in its original formulation that discrete work events trigger affective reactions, both momentary moods and more stable emotions, which accumulate into job attitudes such as job satisfaction. Critically, AET emphasizes that it is the accumulation of recurring negative work events, rather than any single incident, that gradually erodes positive job attitudes. Applied to the present study, mushroom management practices such as being excluded from information and denied participation in decisions constitute recurring negative work events that are expected, consistent with AET, to generate adverse affective reactions and cumulatively diminish nurses’ job satisfaction.

Social identity theory (SIT), developed by Tajfel and Turner [7], proposes that individuals derive part of their self‐concept from membership in social groups, including organizations. When organizational membership is perceived as prestigious, inclusive, and value‐congruent, employees strengthen their identification with the organization. OI, as operationalized by Mael and Ashforth [8], reflects the degree to which an employee perceives oneness with and belongingness to their organization. Applied to nursing, when managers practice mushroom management, signaling distrust and exclusion, nurses’ sense of organizational belonging is undermined, reducing OI and, in turn, depressing job satisfaction.

AET and SIT are not parallel but complementary frameworks operating at different levels of the same psychological process. AET explains the affective pathway: Mushroom management events trigger negative emotions that accumulate into reduced job satisfaction. SIT explains the cognitive‐identity pathway: Exclusionary practices erode OI, which, as a cognitive‐affective bond, further dampens satisfaction. OI thus occupies a pivotal position as a hybrid construct bridging affective and identity‐based processes and provides the theoretical basis for the proposed mediation model.

OI was selected as the mediating variable over other plausible candidates including psychological safety, trust in management, perceived organizational support, and empowerment for both theoretical and empirical reasons. Theoretically, SIT specifically predicts that identity‐related perceptions mediate the effect of organizational practices on work attitudes, and OI is the construct most directly derived from this tradition. Empirically, OI has demonstrated robust meta‐analytic links with job satisfaction and commitment [9] and has been validated in healthcare settings [10]. While psychological safety and trust are important mediators in nursing leadership research [11, 12], they capture affective climate perceptions rather than identity‐based self‐definition. Future research could profitably compare OI alongside these alternatives as competing mediators.

Despite growing interest in knowledge hiding and leadership in healthcare, no empirical study to our knowledge has tested OI as a mediator between mushroom management and job satisfaction among nurses in Türkiye. This gap is noteworthy given that nurses in the Turkish healthcare system often work in hierarchical, high‐pressure environments where managerial opacity can exacerbate stress and reduce motivation [13]. The present study addresses this gap by examining this mediation pathway among nurses working in private hospitals in Diyarbakır, Türkiye.

## 2. Literature Review

### 2.1. Mushroom Management: Conceptual Foundations and Empirical Evidence

Mushroom management describes a leadership style characterized by restricted information sharing, exclusion of employees from decision‐making, and centralized managerial control driven by fear of losing authority [3]. The construct is operationalized across four dimensions as shown in Table 2.

**TABLE 2:** Dimensions of mushroom management.

Dimension	Description
Inadequate information sharing	Managers restrict employees’ access to critical organizational information, limiting awareness of strategic goals and decision‐making processes. This lack of transparency weakens trust and engagement.
Insufficient communication	Organizational communication channels remain weak, leading to misunderstandings, misinformation, and lack of cohesion. Employees often rely on informal channels, which may cause rumors and speculation.
Fear of losing power	Managers withhold information and decision‐making authority due to concerns over losing control and influence, fostering a hierarchical and centralized management style.
Lack of participatory management	Employees are excluded from decision‐making processes, resulting in reduced motivation, job dissatisfaction, and lower organizational commitment.

*Note:* Source adapted from Birincioğlu and Tekin [3].

Although its mechanisms overlap with the knowledge hiding literature, which demonstrates that leader‐driven information restriction undermines psychological safety and collaboration [14, 15], mushroom management is distinct in being a structured managerial style rather than an individual behavioral tendency. Its consequences extend beyond information concealment to encompass broader patterns of exclusion and control that erode organizational trust.

Empirical studies have documented the detrimental effects of mushroom management across sectors. Birincioğlu and Tekin [3] showed its adverse impact on knowledge management in universities. In healthcare and sports, Bozkır and Fidan [13] and Osmanoğlu and Üzüm [16] reported elevated burnout and reduced satisfaction. Çetin [4] found that it increases employee alienation, mediating a negative effect on job satisfaction. Across studies, mushroom management erodes trust [17], amplifies job stress and turnover intentions [18], and weakens supervisory support and performance [5].

However, this body of evidence carries important limitations. Most studies rely on cross‐sectional, self‐report designs, which preclude causal inference and are susceptible to common method bias. A minority of studies, notably Kayapalı [19] and Ilkım and Derin [20], have suggested that information restriction may yield tactical benefits in specific contexts such as the defense industry, where task compartmentalization may reduce cognitive burden. This contextual variability notwithstanding, the weight of evidence points toward net negative outcomes in collaborative, relationship‐intensive workplaces such as hospitals. Furthermore, the existing literature has rarely examined mediating mechanisms, a gap the present study directly addresses.

### 2.2. Mushroom Management and Analogous Leadership Styles in Nursing

Despite the growing literature on mushroom management, its direct investigation in nursing samples remains limited. Nursing presents a particularly high‐stakes context for information‐restrictive leadership: Clinical decision‐making depends on timely, accurate communication; hierarchical structures amplify power asymmetries; and nurses’ emotional labor demands make them especially vulnerable to exclusionary managerial practices.

Systematic reviews consistently identify leadership style as among the strongest determinants of nurses’ job satisfaction and retention [21–23]. Transformational and participative leadership representing the opposite end of the spectrum from mushroom management are robustly associated with higher satisfaction, lower burnout, and stronger commitment among nurses [11, 24]. Supervisory behaviors that suppress voice, restrict autonomy, or undermine trust are linked to elevated stress, reduced psychological safety, and increased turnover intentions [12, 25].

Kılıç [26] examined mushroom management directly in the Turkish healthcare sector, identifying negative implications for service quality and employee outcomes, though without testing a mediation model. Yorgancioglu Tarcan et al. [5] demonstrated that mushroom management reduces perceived supervisory support among hospital employees, negatively affecting performance. Internationally, nurses who perceive managers as withholding critical information report lower trust, weaker unit identification, and reduced satisfaction [25, 27]. In high‐power‐distance contexts such as Türkiye [28], hierarchical norms may intensify the perception and impact of information‐restrictive leadership, making nursing staff particularly susceptible.

### 2.3. Job Satisfaction in Nursing

Job satisfaction, defined as employees’ positive evaluative response to their overall job experience [29], is among the most consequential outcomes in nursing management research, given its established links to care quality, patient safety, and workforce retention. It is important to acknowledge that job satisfaction is a theoretically multidimensional construct [30], encompassing domain‐specific facets such as pay, promotion, supervision, coworkers, and the work itself. The present study employs a validated global measure [29] appropriate for mediation models assessing the cumulative affective impact of a managerial style on overall work attitudes, a widely accepted approach [9]. The limitations of this measurement choice are discussed in Section 6.

### 2.4. OI and Leadership Styles

OI is defined as the perception of oneness with an organization and the incorporation of organizational membership into one’s self‐concept [8]. Meta‐analytic evidence confirms robust positive associations between OI and job satisfaction, organizational citizenship behavior, and reduced turnover intentions [9]. In healthcare settings, OI has been linked to nurses’ commitment, performance, and satisfaction [10]. Leadership that signals respect, openness, and inclusion fosters identification, whereas information withholding and exclusionary practices erode OI by undermining the perceived prestige and inclusiveness of organizational membership [31].

### 2.5. The Mediating Role of OI

The proposed mediation pathway mushroom management ⟶ OI ⟶ job satisfaction is grounded in AET and SIT as elaborated in Section 1. Empirically, mediation via social‐psychological mechanisms is well‐documented in nursing leadership research: Supervisor support mediates transformational leadership effects on satisfaction [11], and psychological safety mediates nurse manager behaviors on commitment [12]. While these studies validate that cognitive‐affective mechanisms transmit leadership influence on nurse outcomes, none specifically tests OI as a mediator in the context of information‐restrictive leadership. This constitutes a substantive gap that the present study addresses.

### 2.6. Research Gap and Contribution

The extant literature reveals three interconnected gaps: (1) No study has tested a mediation model linking mushroom management to job satisfaction via OI; (2) empirical research on mushroom management in nursing in Türkiye remains largely descriptive; and (3) OI has not been empirically examined as a mediator between information‐restrictive leadership and nurse satisfaction. The present study addresses all three gaps by testing a theoretically grounded mediation model, integrating AET and SIT into a coherent dual‐theory framework and providing evidence from the Turkish private healthcare sector where hierarchical norms may amplify the consequences of mushroom management.

## 3. Research Methodology

### 3.1. Purpose of the Study

The aim of this study is to examine the direct and indirect effects of mushroom management on nurses’ job satisfaction, with OI as the mediating variable. The study addresses two key gaps: (1) the lack of empirical evidence on mushroom management in healthcare settings and (2) the absence of mediation models exploring this relationship within the nursing profession in Türkiye.

### 3.2. Population and Sample

According to the General Directorate of Health Information Systems, there are approximately 4770 healthcare workers in Diyarbakır. Only nurses working in private hospitals were considered the target population. Using the sample size formula with a 95% confidence level and a 5% margin of error (*N* = 4770; *t* = 1.96; *p* = *q* = 0.5; *d* = 0.05), the minimum required sample size was calculated as 356 participants.

Data were collected through voluntary participation using a convenience sampling method across private hospitals in Diyarbakır. A total of 317 nurses completed and returned valid questionnaires, yielding a response rate of 89%. The shortfall from the calculated minimum is attributable to the voluntary nature of participation and practical access constraints across multiple institutions. A post hoc power analysis (G∗Power 3.1; *f* ^2^ = 0.18, *α* = 0.05, two predictors) confirmed adequate statistical power (1 − *β* = 0.97) for the analyses conducted. This is discussed further in Section 6.

### 3.3. Ethical Considerations

The study received ethical approval from the Fırat University Non‐Interventional Clinical Research Ethics Committee (Approval No: 2025/06, Date: 13.03.2025). Prior to participation, all respondents were informed about the study’s aims, procedures, and voluntary nature. Written informed consent was obtained, and confidentiality was assured by anonymizing all responses. Participants were informed that they could withdraw at any stage without consequences.

### 3.4. Data Collection Instruments

Three validated scales were used. All scales use a five‐point Likert format (1 = *strongly disagree* to 5 = *strongly agree*), with higher scores indicating greater levels of the measured construct.

Mushroom Management Scale: It was developed and validated in the Turkish context by Birincioğlu and Tekin [3]; this scale comprises 19 items across four subdimensions: inadequate information sharing, insufficient communication, fear of losing power, and lack of participatory management. Higher scores reflect greater managerial opacity. The scale was originally developed with Turkish samples, ensuring linguistic and cultural appropriateness. Cronbach’s alpha in the current study = 0.91.

Job Satisfaction Scale: The five‐item short form developed by Judge and Klinger [29], adapted to Turkish by Keser and Bilir [32], assesses global job satisfaction. Higher scores indicate greater overall satisfaction. This global measure captures nurses’ general evaluative response to their work and is appropriate for mediation studies assessing affective work outcomes as a unitary construct. Cronbach’s alpha = 0.85.

Organizational Identification Scale: It was developed by Mael [33] and adapted to Turkish by Tüzün and Özdoğan [34]; this six‐item single‐factor scale measures employees’ sense of oneness with their organization. Higher scores reflect stronger identification. The scale has been widely used in organizational and health services research [10]. Cronbach’s alpha = 0.79.

#### 3.4.1. Validity and Reliability

Confirmatory factor analysis (CFA) was performed using AMOS 24.0. The three‐factor measurement model demonstrated good fit (see Table 3 in Findings). All standardized factor loadings were significant (*p* < 0.001) and above 0.50. Average variance extracted (AVE) exceeded 0.50 for all constructs, supporting convergent validity. Discriminant validity was confirmed using the Fornell and Larcker [36] criterion: the square root of each construct’s AVE exceeded the interconstruct correlations in all cases.

**TABLE 3:** CFA model fit indices.

Model	*χ* ^2^/d*f*	CFI	TLI	RMSEA	SRMR	Threshold
Three‐factor model	2.21	0.95	0.94	0.062	0.047	
Recommended	< 3.00	≥ 0.95	≥ 0.90	< 0.08	< 0.08	[35]

Abbreviations: CFI, comparative fit index; RMSEA, root mean square error of approximation; SRMR, standardized root mean square residual; TLI, Tucker–Lewis index.

### 3.5. Common Method Bias Control

Several procedural and statistical steps minimized common method variance (CMV). Procedurally, anonymity and confidentiality were assured, negatively worded items were included, items were presented in randomized order, and participation was voluntary. Statistically, Harman’s single‐factor test was performed (first factor = 31.7% of variance, below the 50% threshold). However, consistent with Podsakoff et al. [37], we acknowledge that this test is insufficient as a standalone diagnostic. A CFA marker variable check was additionally conducted, with fit indices not changing substantially, suggesting CMV is unlikely to have severely distorted findings. Future research should consider ULMF models for more rigorous CMV control.

### 3.6. Data Analysis

All analyses were conducted using IBM SPSS Statistics 23.0 and AMOS 24.0. Descriptive statistics, Pearson correlations, and reliability analyses were performed in SPSS. CFA and SEM fit indices were evaluated in AMOS 24.0. Mediation analysis was conducted using Hayes’ PROCESS macro (Model 4) in SPSS with 5000 bootstrap samples at a 95% confidence interval, controlling for demographic characteristics (age, gender, marital status, education, and work experience).

### 3.7. Research Model and Hypotheses

 H1: Mushroom management is negatively associated with OI.
 H2: Mushroom management is negatively associated with job satisfaction.
 H3: OI is positively associated with job satisfaction.
 H4: OI mediates the relationship between mushroom management and job satisfaction.

## 4. Findings

### 4.1. Demographic Characteristics of Participants

Table 4 presents the demographic profile of the 317 participating nurses. The sample was predominantly female (78.9%), consistent with the gender composition of the nursing profession in Türkiye. The majority of participants were in the 22–31 age group (56.8%), held a bachelor’s degree (85.2%), were married (60.6%), and had 1–5 years of work experience (56.8%). This profile reflects the relatively young and early‐career composition of nursing staff in private hospitals in the region.

**TABLE 4:** Demographic characteristics of participants (*N* = 317).

Variable	*n*	%
Age		
22–31	180	56.8
32–39	101	31.9
40 and above	36	11.4
Gender		
Female	250	78.9
Male	67	21.1
Marital status		
Married	192	60.6
Single	125	39.4
Education level		
Bachelor’s degree	270	85.2
Postgraduate degree	47	14.8
Work experience		
1–5 years	180	56.8
5–10 years	101	31.9
11 years and above	36	11.4
Total	317	100.0

*Note:* Percentages may not sum to 100.0 due to rounding.

### 4.2. Descriptive Statistics and Correlations

Table 5 presents the descriptive statistics, reliability coefficients, and Pearson correlations. The mean mushroom management score (*M* = 3.41, SD = 0.72) was above the scale midpoint, indicating that nurses perceived moderate‐to‐high levels of information‐restrictive leadership. OI (*M* = 2.84, SD = 0.68) was below the midpoint, and job satisfaction (*M* = 3.12, SD = 0.74) was near the midpoint, suggesting relatively constrained organizational belonging and satisfaction in this sample. Mushroom management was significantly and negatively correlated with both OI (*r* = −0.46, *p* < 0.001) and job satisfaction (*r* = −0.39, *p* < 0.001). OI showed a significant positive correlation with job satisfaction (*r* = 0.52, *p* < 0.001).

**TABLE 5:** Descriptive statistics, reliabilities, and correlations.

Variable	Mean	SD	*α*	Min–Max	1	2
1. Mushroom management	3.41	0.72	0.90	1–5	—	—
2. Organizational identification	2.84	0.68	0.79	1–5	−0.46^∗∗∗^	—
3. Job satisfaction	3.12	0.74	0.85	1–5	−0.39^∗∗∗^	0.52^∗∗∗^

*Note:* ^∗∗∗^ *p* < 0.001. All scale scores range from 1 to 5.

### 4.3. CFA

A three‐factor CFA model was tested in AMOS 24.0. As shown in Table 3, the model demonstrated good fit across all indices. All standardized factor loadings ranged from 0.52 to 0.81 (*p* < 0.001), exceeding the 0.50 threshold. AVE values exceeded 0.50 for all constructs, supporting convergent validity. The Fornell and Larcker [36] criterion confirmed discriminant validity.

### 4.4. Regression Analysis

Table 6 presents the direct effects. Mushroom management had a significant negative effect on OI (sandardized *β* = −0.46, SE = 0.05, *t* = −9.20, *p* < 0.001), supporting H1. It also had a significant negative direct effect on job satisfaction (standardized *β* = −0.21, SE = 0.06, *t* = −3.50, *p* = 0.001), supporting H2. OI positively predicted job satisfaction (standardized *β* = 0.48, SE = 0.05, *t* = 9.60, *p* < 0.001), supporting H3. The effect of mushroom management on OI was notably large, indicating that perceived managerial opacity is strongly associated with nurses’ diminished sense of organizational belonging.

**TABLE 6:** Regression analysis results.

Path	Standardized *β*	SE	*t*	*p*
Mushroom management ⟶ organizational identification	−0.46	0.05	−9.20	< 0.001
Mushroom management ⟶ job satisfaction	−0.21	0.06	−3.50	0.001
Organizational identification ⟶ job satisfaction	0.48	0.05	9.60	< 0.001

*Note:* Standardized *β* = standardized regression coefficient. All models controlled for demographic variables.

Abbreviation: SE, standard error.

### 4.5. Mediation Analysis

A mediation model was tested using PROCESS macro (Model 4) with 5000 bootstrap samples. As shown in Table 7, the indirect effect of mushroom management on job satisfaction via OI was statistically significant (standardized *β* = −0.22, BootSE = 0.04, 95% CI [−0.31, −0.14]), as the confidence interval did not include zero. The direct effect remained significant after including the mediator (standardized *β* = −0.21, *p* = 0.001), confirming partial mediation and supporting H4. The indirect effect accounted for 51% of the total effect (standardized *β* = −0.43), indicating that OI is the primary, though not exclusive, mechanism through which mushroom management is associated with lower job satisfaction.

**TABLE 7:** Mediation analysis results (bootstrap, 5000 samples).

Effect type	Standardized *β*	SE	95% CI (LL, UL)	*p*
Direct effect (MM ⟶ JS)	−0.21	0.06	[−0.33, −0.09]	0.001
Indirect effect (MM ⟶ OI ⟶ JS)	−0.22	0.04	[−0.31, −0.14]	—
Total effect	−0.43	0.06	[−0.55, −0.31]	< 0.001

*Note:* Standardized *β* = standardized regression coefficient. Bootstrap CI not including zero indicates significant indirect effect.

Abbreviations: JS, job satisfaction; MM, mushroom management; OI, organizational identification.

### 4.6. Summary of Findings

All four hypotheses were supported. Mushroom management was significantly and negatively associated with both OI (H1: standardized *β* = −0.46) and job satisfaction (H2: standardized *β* = −0.21). OI was positively associated with job satisfaction (H3: standardized *β* = 0.48). Mediation analysis confirmed partial mediation via OI (H4: indirect standardized *β* = −0.22, 95% CI [−0.31, −0.14]), accounting for 51% of the total effect.

## 5. Discussion

This study examined the relationships between mushroom management, OI, and job satisfaction among nurses in private hospitals in Türkiye. All four hypotheses were supported, and the findings offer both theoretical and practical insights into how information‐restrictive leadership shapes nurses’ psychological and affective work experiences.

### 5.1. Hypothesis Evaluations

H1 was supported (standardized *β* = −0.46, *p* < 0.001): Mushroom management substantially erodes nurses’ OI. The magnitude of this effect, explaining approximately 21% of variance in OI, indicates that perceived managerial opacity constitutes a substantive threat to nurses’ sense of organizational belonging. H2 was supported (standardized *β* = −0.21, *p* = 0.001): Mushroom management is directly associated with lower job satisfaction, independent of the mediated pathway. H3 was supported (standardized *β* = 0.48, *p* < 0.001): Nurses with stronger OI report meaningfully higher job satisfaction, consistent with meta‐analytic estimates [9]. H4 was supported: the indirect effect (standardized *β* = −0.22, 95% CI [−0.31, −0.14]) accounted for 51% of the total effect, confirming partial mediation.

### 5.2. Mushroom Management and OI

The finding that mushroom management substantially erodes OI is consistent with SIT [7], which predicts that leadership practices signaling exclusion and distrust undermine employees’ perceived oneness with the organization. In the specific context of Diyarbakır’s private hospitals, this effect may be amplified by structural features: Private hospitals in Türkiye tend to operate under strong centralized ownership, with managers facing pressure to maximize productivity while minimizing labor autonomy. In such environments, mushroom management behaviors are not merely individual managerial choices but may reflect institutionalized governance norms. For nurses, whose professional identity is closely tied to organizational belonging, these practices are particularly identity‐threatening.

Practically, the sample’s mean mushroom management score (*M* = 3.41) is above the scale midpoint, indicating that the majority of participating nurses are experiencing levels of managerial opacity that the model predicts will meaningfully suppress OI. This is a signal of a widespread organizational climate problem rather than a marginal statistical finding.

### 5.3. Mushroom Management, OI, and Job Satisfaction

The partial mediation finding indicates that OI accounts for 51% of the total effect. This extends prior nursing leadership research which has established social‐psychological mediators such as supervisor support [11] and psychological safety [12] by demonstrating that an identity‐based mechanism operates alongside affective climate variables. The remaining direct effect (standardized *β* = −0.21) suggests additional mechanisms consistent with AET’s concept of affect‐driven responses: Mushroom management events may directly trigger discrete negative reactions frustration, perceived powerlessness that accumulate into reduced satisfaction independently of identity processes.

In practical terms, the sample’s job satisfaction mean (*M* = 3.12) combined with high mushroom management exposure and below‐midpoint OI signals a workforce at elevated risk for turnover, a combination that is strongly predictive of intent to leave in the nursing literature [21, 22].

### 5.4. Theoretical Contributions

This study makes three distinct theoretical contributions. First, it extends SIT to nursing management research by demonstrating that OI mediates the effect of mushroom management on job satisfaction, identifying managerial opacity and exclusion as antecedents of OI erosion in clinical settings. Second, it advances AET–SIT integration by showing that the two frameworks operate at distinct but complementary levels: AET accounts for the direct affective pathway (H2), while SIT accounts for the identity‐mediated pathway (H1 and H4). OI is positioned as a hybrid cognitive‐affective construct that bridges both theories. Third, the study moves the mushroom management literature from descriptive documentation to theoretical explanation by specifying, for the first time in a nursing sample, OI as the psychological mechanism linking this managerial style to job satisfaction.

### 5.5. Cultural and Contextual Considerations

The Turkish cultural context constitutes both a boundary condition and an explanatory factor for the observed findings. Türkiye scores relatively high on Hofstede’s [28] power distance dimension, reflecting societal acceptance of hierarchical authority. In high‐power‐distance cultures, employees are less likely to challenge managerial information withholding, and the expectation that managers control information flows may be more deeply institutionalized. This cultural norm may simultaneously normalize mushroom management behaviors and limit nurses’ capacity to resist their identity‐threatening effects.

The private hospital context in Diyarbakır further shapes these dynamics. Private hospitals may exhibit stronger managerial centralization than large urban teaching hospitals or public institutions. The relative scarcity of alternative nursing employment in this regional context may also reduce exit options, potentially intensifying the affective impact of sustained mushroom management exposure. These contextual specificities suggest that the effect sizes observed may be amplified relative to more structurally empowering settings, and cross‐context comparative research should examine these boundary conditions.

### 5.6. Practical Implications for Nursing Management

The findings carry concrete implications for nursing management that extend beyond generic transparency recommendations. First, healthcare institutions should implement structured information‐sharing protocols, guaranteeing nurses access to organizational decisions affecting their units including mandatory preshift briefings on organizational updates, nurse representation on hospital management committees, and digital dashboards providing real‐time departmental data. These measures directly address the inadequate information sharing and a lack of participatory management dimensions of mushroom management.

Second, leadership development programs should explicitly address mushroom management’s behavioral indicators. Training should help managers recognize the identity consequences of information withholding and develop participatory decision‐making skills through role‐play scenarios modeled on the specific dimensions of mushroom management.

Third, institutions should invest in identity‐affirming practices including professional recognition systems, interdepartmental mentoring programs, and shared governance councils to strengthen nurses’ organizational belonging independently of immediate managerial behaviors. In the Diyarbakır private hospital context, where hierarchical norms may limit spontaneous voice, formal structural channels for nurse input are especially critical.

Fourth, periodic organizational climate surveys measuring mushroom management, OI, and satisfaction using the validated Turkish‐language scales from this study [3, 32, 34] would allow administrators to identify at‐risk units early and target managerial development resources accordingly.

## 6. Limitations and Future Research Directions

### 6.1. Cross‐Sectional Design and Causal Inference

The most fundamental limitation is the cross‐sectional design, which precludes definitive causal inference. Although the hypothesized causal sequence is theoretically grounded, the simultaneous measurement of all variables means that alternative causal orderings cannot be ruled out empirically. Reverse causation, whereby nurses with lower job satisfaction perceive their managers as more information‐restrictive, is plausible. Furthermore, the simultaneity of measurement in cross‐sectional mediation models may conflate theoretically sequential processes, potentially inflating indirect effect estimates [38]. Future research should employ longitudinal designs with multiple measurement waves and experimental or quasiexperimental designs to establish temporal ordering and causal relationships.

### 6.2. Sample Characteristics and Generalizability

The sample was drawn exclusively from nurses in private hospitals in Diyarbakır. Private hospitals in Türkiye tend to operate under stronger centralized ownership and profit‐driven governance than public or university hospitals, which may shape managerial practices differently. Nurses in public hospitals, governed by Ministry of Health regulations with civil service employment protections, may experience and respond to mushroom management differently. Regional labor market conditions in Diyarbakır including limited alternative nursing employment may intensify the affective impact of mushroom management. The findings should not be directly generalized to public hospitals, primary healthcare institutions, or nursing workforces in other Turkish cities or countries with different cultural and organizational norms. Future research should replicate the model across diverse institutional and cultural settings.

### 6.3. Sample Size and Statistical Power

Although the a priori calculation indicated a minimum of 356 participants, the final sample was 317. Post hoc power analysis (G∗Power 3.1; *f* ^2^ = 0.18, *α* = 0.05, two predictors) confirmed adequate power (1 − *β* = 0.97) for the primary analyses [39]. However, future studies employing moderated mediation or multigroup designs should ensure larger samples to guarantee parameter stability and reliable confidence interval estimation.

### 6.4. Measurement Limitations

Three measurement limitations are noteworthy. First, the global job satisfaction scale [29] does not capture facet‐specific variation in satisfaction. Future studies employing multidimensional instruments (e.g., Minnesota Satisfaction Questionnaire and Job Descriptive Index) would enable more nuanced examination of how specific mushroom management dimensions affect specific satisfaction facets. Second, all scales were administered simultaneously in a single survey, creating CMV potential. While Harman’s test and a CFA marker variable check were employed, Harman’s test is recognized as insufficient in isolation [37]; future research should employ ULMF models or independent criterion sources. Third, the Mushroom Management Scale [3] has not been specifically adapted for or validated in clinical nursing populations; future research should examine its factorial invariance across nursing subsamples.

### 6.5. Structural Equation Modeling Limitations

The mediation model was tested using PROCESS macro with observed composites rather than latent variables, meaning measurement error in predictors and outcomes is not modeled. Observed‐variable mediation may yield attenuated or biased estimates when scale reliabilities are imperfect, a consideration relevant here given the OI scale’s alpha (0.79) at the lower bound of acceptability. Future studies with larger samples should consider full latent variable SEM in AMOS or Mplus for more rigorous mediation testing. Cross‐lagged panel SEM would additionally address the temporal precedence limitation.

## 7. Conclusion

This study provides empirical evidence that mushroom management is associated with lower OI and job satisfaction among nurses in private hospitals in Diyarbakır, Türkiye, with OI serving as a significant partial mediator. These findings are among the first to empirically test this mediation pathway in a nursing sample, advancing the theoretical integration of AET and SIT in explaining how managerial opacity translates into diminished nurse well‐being.

The results underscore the critical importance of transparent communication, participatory leadership, and identity‐affirming management practices for fostering nurse engagement and satisfaction. Healthcare organizations, particularly in high‐power‐distance cultural contexts and centralized private hospital settings, should prioritize structural reforms that guarantee nurse access to organizational information, create formal channels for nurse voice, and build leadership competencies that recognize and counteract the identity‐threatening effects of mushroom management.

## Funding

No funding was received for this manuscript.

## Conflicts of Interest

The authors declare no conflicts of interest.

## Data Availability

The data that support the findings of this study are available from the corresponding author upon reasonable request.
